# Hygiene in medical education – Increasing patient safety through the implementation of practical training in infection prevention

**DOI:** 10.3205/zma001223

**Published:** 2019-03-15

**Authors:** Annika Richter, Iris F. Chaberny, Alexander Surikow, Bettina Schock

**Affiliations:** 1 University Hospital Leipzig, Centre for Infection Medicine (ZINF), Institute of Hygiene, Hospital Epidemiology and Environmental Health, Leipzig, Germany

**Keywords:** patient safety, learning objectives, teaching, nosocomial infections, hygiene

## Abstract

**Objective: **Insufficient hygiene knowledge increases the risk of hospital-acquired infections through insufficient compliance and therefore poses a potential risk to patient safety. Therefore in 2015 the teaching project “OT Training” was introduced at the Faculty of Medicine (MF) Leipzig and a restructuring of the series of lectures and practical training on the topic of “Hygiene” was developed and integrated in the medical study curriculum.

**Methodology: **The “OT Training” in the pre-clinical component and the didactic restructuring of the hygiene workshops in the hospital semester were comprehensively developed by means of the currently applicable learning objective catalogues and have already been tested in existing teaching (per year N=320 students; 2015-17: N= 960). The “OT Training” and the series of lectures and practical training are evaluated externally by the Faculty of Medicine. In addition a self-developed questionnaire (for “OT Training”) and an internal evaluation (for practical stations as part of the practical training series) were used.

**Results: **Overall the “OT Training” was evaluated as “very good” (N=492; RR=51%). Alongside the high importance of hygiene in the hospital and operating area (Overall_hospital_=97% and Overall_Operative area_=98%) the salient feature of hygiene for self-protection and in particular for patient safety was also recognised at an early stage. Through the series of lectures and practical training which were also evaluated positively, the self-reported level of knowledge and the importance of hygiene for the students improved significantly (level of knowledge M_before_=2.8 vs. M_after_=3.9; p>0.000; importance M_before_=3.3 vs. M_after_=4.2; p>0.000; 5 point Likert scale; t-Test).

**Conclusion: **Hygiene errors constitute a potential risk to patients. Consequently the early and continuous focus on hygiene in student education makes a contribution to increasing patient safety in the healthcare sector.

## Zusammenfassung

**Zielsetzung:** Mangelnde Hygienekenntnisse erhöhen durch mangelnde Compliance das Risiko von Krankenhausinfektionen und stellen somit eine potenzielle Gefahr für die Patientensicherheit dar. Daher wurde an der Medizinischen Fakultät (MF) Leipzig 2015 das Lehrprojekt „OP-Training“ eingeführt sowie eine Umstrukturierung der Vorlesungs- und Praktikumsreihe „Hygiene“ ausgearbeitet und in das Curriculum des Humanmedizinstudiums integriert.

**Methodik: **Das „OP-Training“ in der Vorklinik und die didaktische Umstrukturierung der Hygiene-Veranstaltungen im Kliniksemester sind mittels aktuell geltender Lernzielkataloge umfassend ausgearbeitet und bereits in der regulären Lehre (pro Jahr N=320 Studierende; 2015-17: N= 960) erprobt. Sowohl das „OP-Training“ als auch die Vorlesungs- und Praktikumsreihe werden durch die MF extern evaluiert. Zusätzlich wurde ein selbstentwickelter Fragebogen (für „OP-Training“) und eine interne Evaluation (für Übungsstationen der Praktikumsreihe) eingesetzt.

**Ergebnisse:** Das „OP-Training“ wird in der Gesamtheit mit „sehr gut“ evaluiert (N=492; RQ=51%). Neben dem hohen Stellenwert der Hygiene im Klinik- und OP-Bereich (Gesamt_Klinik_=97% und Gesamt_OP-Bereich_=98%) wurde ebenso die Besonderheit der Hygiene als Eigenschutz und vor allem als Patientenschutz frühzeitig erkannt. Durch die ebenfalls positiv evaluierte Vorlesungs- und Praktikumsreihe verbesserte sich der selbstberichtete Kenntnisstand und der Stellenwert der Hygiene der Studierenden signifikant (Kenntnisstand M_vorher_=2,8 vs. M_nachher_=3,9; p>0,000; Stellenwert M_vorher_=3,3 vs. M_nachher_=4,2; p>0,000; 5-Stufige Likert-Skala; t-Test).

**Schlussfolgerung: **Fehler in der Hygiene stellen eine potenzielle Patientengefährdung dar. Somit leistet die frühzeitige und kontinuierliche Fokussierung der Hygiene in der studentischen Ausbildung einen Beitrag zur Erhöhung der Patientensicherheit im Gesundheitswesen.

## Introduction

Nosocomial infections (NI) constitute a serious complication for patients as part of their stay in hospital and are associated with a longer stay in hospital, higher mortality and increased costs for the healthcare sector [1], [2], [3], [4]. According to the results of the third national prevalence study in Germany the overall prevalence of NI was 4.6% [5]. Alongside lower respiratory infections, postoperative wound infections are the most common NI, which past prevalence studies have also shown along with those of a university hospital, in which the overall prevalence was 11.2% [6], [7]. The safety of the patient is the focal point of every quality-oriented healthcare service [8]. Hygiene, as a subject of preventative medicine, is dedicated amongst other aspects to the avoidance of infections and the containment and spread of pathogens with an extended resistance spectrum while also providing an intensive explanation of these aspects. Insufficient hygiene knowledge can potentially lead to the inadequate observance of infection prevention measures and consequently to the increase of hospital infections [9], which are regarded as complications and therefore constitute a risk to patients. The competence requirement of medical students has been developed as part of the National Competence Based Catalogue of Learning Objectives in Medicine (NKLM [http://www.nklm.de). In the process the relevance of the competence “Skills in prevention” is also emphasised, so that this focal point increasingly becomes more important in the teaching of students. In joint recommendations of the Association of German Faculties (MFT) and the Commission for Hospital Hygiene and Infection Prevention (KRINKO) at the Robert Koch Institute for the education of hospital hygiene at German universities it is emphasised that it is not only the theoretical teaching of hospital hygiene measures which is important, but also that the practical relevance is established and measures are trained to prevent infection [10]. This aspect has also been taken up internationally by the World Health Organisation (WHO), whereupon the requirement of changed teaching methods and modules becomes apparent, so that the students’ level of knowledge for the prevention of infections and dealing safely with the prevention measures can be improved [11], [12], [13]. Furthermore conveying up-to-date hygienic teaching content at German universities, e.g. for the operating area, is insufficiently integrated in the medical study curriculum [10]. It is essential to initiate in particular the strengthening of the importance of hygiene and therefore inextricably the connection with patient safety, among other reasons, because it is known that postoperative wound infections are amongst the most common types of nosocomial infection in German hospitals [6], [7]. Infection prevention and the necessary intensive educational measures in the form of hygiene training courses take place predominantly in everyday clinical practice for medical and nursing staff. 

Therefore an innovative contribution could be to integrate prospective medical practitioners at the beginning and during medical study in the process sequence of infection prevention and its importance for patient safety and also practise the prevention of infections. For this reason and with the creation of the Chair in the subject hygiene with the focal point hospital hygiene at the Faculty of Medicine in Leipzig (MF), in 2015 the teaching project Introduction to Clinical Medicine (ICM) – “OT Training” was introduced and a restructuring of the already existing hygiene series of lectures and practical training was developed and since 2017 this new project has been integrated in the existing curriculum. 

## Project description

**Figure 1:**
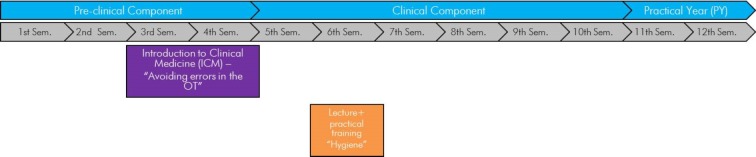
Workshops for hygiene and hospital hygiene in the medical curriculum, Faculty of Medicine Leipzig

The medical study curriculum at the Faculty of Medicine at Leipzig University currently includes two sequential workshops in the subject “Hygiene and Hospital Hygiene” (see figure 1 (Fig. 1)). The total number of students per semester at the Faculty of Medicine varies between 300 and 320 students, who as part of their medical study are currently taught the specialist field of hygiene in the pre-clinical component and at the clinical component. In the second academic year the students receive as part of Introduction to Clinical Medicine (ICM) – in the course “OT Training – avoiding errors in the OT” an initial insight into the specialist field. In 2015 this course was integrated as a teaching project in the workshop series Introduction to Clinical Medicine (ICM) and the main focus is on practical training, which is based on the criteria of the National Competence Based Catalogue of Learning Objectives in Medicine (NKLM). In the third academic year there is first of all a comprehensive transfer of knowledge in a series of lectures, which forms the theoretical basis for subsequent hygiene practical training. The hygiene practical training, consisting of three main topics (nosocomial infections, environmental hygiene and outbreak management), was restructured in 2017 in accordance with the guidelines of the National Competence Based Catalogue of Learning Objectives in Medicine (NKLM) and the requirements of the learning and competence objectives for the area of hygiene. While the practical training before 2017 had more of a seminar character in its practical implementation (theoretical processing of case studies) and a detailed theoretical introduction took place at the beginning of the workshop, the main focus is now placed on practically relevant, practical training. After a ca. 20 minute presentation the students have the opportunity to be practically active for at least 90 mins, before a ca. 20 minute joint debriefing brings the practical training to an end. The time scale for each practical workshop has remained constant.

As part of the implementation of the teaching project “OT Training – avoiding errors in the OT” and the didactic restructuring of the series of lectures and practical training “hygiene”, comprehensive developments to the currently applicable learning objectives catalogues have taken place in the teaching team of the Institute of Hygiene, Hospital Epidemiology and Environmental Health. In the process the orientation was based on the learning and competence objectives for the area of hygiene of the National Competence Based Catalogue of Learning Objectives in Medicine (NKLM) [10]. Based on the fact that (hand) hygiene in basic hygienic understanding is intrinsically indispensable for patient safety, the adopted learning and competence objectives were also compared with the learning objective catalogues for patient safety (see table 1 (Tab. 1) [http://www.nklm.de], [13], [14], [15]). As part of the following teaching descriptions, table 1 (Tab. 1) and the respective comparison of the learning objectives of various learning objective catalogues are used.

### Teaching description Introduction to Clinical Medicine (ICM) course “OT Training – avoiding errors in the OT”

The students (N=ca. 320) are divided up for the Introduction to Clinical Medicine (ICM) course so that one half of the students in the 3rd semester attend the workshop and the remaining students attend it in the 4^th^ semester. Conceptually the workshop is carried out in an interactive practical form with a theoretical seminar character. In the 15 minute theoretical introduction the students are given a historic overview of the subject, indication-specific explanations of hygienic and surgical hand disinfection, an introduction to the relevance of postoperative wound infections on the basis of a memorable case study and structural background information about operating areas (structural/spatial separation, scrubs clothing etc.) and behaviour which must always be observed. In addition there is the introduction to the guidelines for the sluice room which provides the practical transition to “Avoiding errors in the OT”. In the practical section of the workshop (ca. 70 mins) the students learn the correct hygienic hand disinfection with fluorescent hand disinfectant. The implementation of hygienic and surgical hand disinfection by the students in accordance with the guidelines is checked with the aid of a black-light lamp. Subsequently the students dress themselves for the operating area and in the process learn in particular to be dressed in a correct sterile manner and how to behave in the operating theatre. There is plenty of scope to resolve any questions or uncertainties of the students, once again demonstrate the hygienic procedure by the tutor and the students therefore receive the necessary instruction, which is often absent in clinical clerkships or practical employment in operating areas. The primary learning and competence objective of the “OT Training” is above all that the students of the 3^rd^/4^th^ semester are able to behave appropriately in the operating area following the teaching unit. In addition through the workshop they are also able to recognise typical sources of error in the operative area and know how to avoid them in accordance with patient safety. Overall the workshop helps to take away the initial uncertainty that the students experience in the operating area and they are therefore able to pay greater attention to appropriate behaviour.

#### Teaching description Hygiene (lecture and practical trainings)

In the third academic year as part of the series of lectures there is first of all a comprehensive transfer of knowledge which forms the theoretical basis for the subsequent practical trainings (see table 1 (Tab. 1)). The series of lectures remains unchanged covering six thematically sequential workshops (introduction to hygiene, disinfection & sterilisation, prevention of selected infections, focus on multi-resistant pathogens (MRP), environmental hygiene as well as vaccination and travel). The 90 minute lectures pursue the primary learning objective of providing the students with an overview of the hygiene and safety culture and the resultant patient safety. With the aid of the workshop the students are able to assess which proportion of NI is avoidable and establish the most important measures for the prevention of NI. Through the workshop a connection is made between the recognition of frequent barriers to hygiene and their influence on patient safety [16]. In a subsequent revision class the content of all workshops is repeated and also the connection with the patient safety measures in accordance with the learning objective catalogue patient safety for medical study [16] and the overriding objective of the avoidance of NI is created. Through the practical trainings, which take place at the same time, the students are able to directly apply the theoretical learning objectives by practising a manner of working which prevents infections. 

The hygiene practical training, consisting of three main topics (nosocomial infections, environmental hygiene and outbreak management), was restructured in 2017 from a workshop with a seminar character to a practical workshop. It did not involve a thematic refocussing with regard to content, instead the didactic teaching of the content was completely revised. The overriding objective of the restructuring was therefore to design the workshop in a manner so it was more comprehensible and more attractive for the students from a hygienic perspective, but also to achieve lasting effects with regard to the routine implementation of hygiene measures. In order to do justice to the practical character, at the beginning of the class the ca. 30 students are divided into groups at random. In small groups of five to six students various practical stations are worked on. The sequence is identical in all three units. There is a short theoretical repetition and connection with the content of the lectures, a division into small groups, a time frame for working on each station (between 15-20 mins per station), when the time expires the group moves to the next practical station, upon completion of all practical training for working in a manner which prevents infections there is a debriefing of the individual stations and the correct hygienic approach is once again demonstrated by the students and evaluated by the tutor. 

In the first practical training unit “Nosocomial Infections” the students go through six practical stations in rotation:

1. Station for surgical and hygienic hand disinfection,
2. Station for campaigns for infection prevention,
3. Station with a clinical case about postoperative wound infections and the importance for the patient,
4. Station for dealing with medication,
5. Station for the correct use and practice with sterile gloves and
6. dealing with personal protective equipment.

The second practical workshop “Environmental Hygiene” covers five practical stations:

1. Station for taking a drinking water sample,
2. Station for an airborne germ sample using the example of mould,
3. Station for the interpretation of findings of an airborne germ sample and
4. Station for the interpretation of findings of a drinking water sample for Legionella and the risk assessment for patient safety and
5. Station for notifiable diseases.

In the final practical training unit the students go through four comprehensive practical units with the focal point “outbreak management”:

1. Station for the conception and expectations of further training covering hygiene,
2. Station for vaccination difficulties and initiative for vaccination behaviour,
3. Station about isolation measures and MRP in relation to personal and patient safety and
4. Station with a virtual station plan and various outbreak scenarios.

Following the series of workshops in the 6th semester the students participate in a written multiple-choice examination. The content of the examination covers the topics of Microbiology, Virology, Immunology and Hygiene in equal parts (10 questions per specialist field). The hygiene questions, which are newly created each year in accordance with the guidelines of the Institute for Medical and Pharmaceutical Examination Questions (IMPP), cover topics from the series of lectures and practical training.

#### Evaluation possibilities of workshops

For the Introduction to Clinical Medicine (ICM) workshop “OT Training – avoiding errors in the OT” (since 2015) and the series of lectures and practical training on the topic of “Hygiene” (since 2017) external teaching evaluations are carried out by the Department of Teaching of the Faculty of Medicine Leipzig with the aid of the software EvaSys® (https://www.evasys.de/evasys.html). Through the implementation of the Introduction to Clinical Medicine (ICM) teaching project in medical study a short questionnaire (see attachment 1 ) was also developed by the teaching team and following an internal pretest used at the beginning of the workshop. This questionnaire deals with the self-reported importance of hospital hygiene and infection prevention from the point of view of the students as part of their study, principally in everyday clinical practice and in the operative area and regarding existing prior knowledge and expectations through the participation in the OT training.

As part of the practical training series on the topic of “hygiene” the students were asked for, alongside the conventional external evaluation, a detailed internal evaluation of all practical stations with regard to the relevance of content and quality of the practical implementation and their assessment of the importance of hygiene (before and after the workshop) and their own level of knowledge (before and after the workshop) (see attachment 2 ).

## Results

### Evaluation of the Introduction to Clinical Medicine (ICM) course “OT Training – avoiding errors in the OT”

With the aid of table 2 (Tab. 2) it is clear that over the period of 2015 to 2017 the majority of the students of the respective 3^rd^/4^th^ semester stated beforehand that they had average to low prior hygienic knowledge in the area of infection prevention (Overall=72%; 2015=79%; 2016=64%; 2017=73%). They also stated that after the workshop they would like to acquire practical skills in the area of infection prevention (Overall=78%). 

At the same time the importance of infection prevention in everyday clinical practice and in the operating area was rated as very high by the students (Overall_ Everyday clinical practice_=97% and overall _Operating area_=98%). As part of study the importance in comparison with the hospital and operating areas was evaluated as being lower (overall=63%). Following the first run of the teaching project in the winter semester 2015, the workshops were evaluated positively by the students via EvaSys^®^ – overall rated as “good” (mark 1.6; n=75). Following the second run it was possible to achieve an improvement in the overall evaluation so that the final evaluation for the Introduction to Clinical Medicine (ICM) course in 2016 was 1.4 = “very good” (n=259). In the third year it was possible to maintain a very good evaluation (2017 mark 1.5; n=158). Through the free text analysis after the Introduction to Clinical Medicine (ICM) workshops over the course of the year it emerged that a large number of the students evaluated the practical component as being extremely important. Furthermore through the free text analysis it was possible to identify that the students would like to have hygiene with the focus “Repetition of the OT training” as a longitudinal study topic, as the trained actions would become automatic actions in the future, which are subsequently implemented routinely to prevent infection and should be repeatedly practised. The special feature of focussing on hygiene not only as self-protection, but also from a patient perspective the safety culture at the beginning of the study was also described as positive. 

#### Evaluation of the series of lectures and practical training on the topic of “hygiene”

In the internal evaluation of the practical training series in 2017 189 students took part (response rate (RR)= 59%; In total 320 students). In their 2^nd^ academic year in 2016 this student cohort took part in the “OT training” and subsequently in 2017 the series of lectures and practical training. Through the internal evaluation it was possible to record from the students, among other aspects, the extent to which their self-reported hygienic level of knowledge and their assessment of the personal importance of infection prevention had changed through the series of workshops. In the process it was shown that the level of knowledge as well as the importance of hygiene improved significantly (level of knowledge M_before_=2.8 vs. M_after_=3.9; p>0.000 and importance of hygiene M_before_=3.3 vs. M_after_=4.2; p>0.000; 5 point Likert scale 1=very low to 5=very high; t-Test).

Through the individual evaluations of the 15 practical stations from the three practical training units it is clear that overall the stations were largely well received by the students in terms of relevance (Range= 3.15-4.38) and the quality of the practical implementation (Range=3.14-4.49) (cf. table 3 (Tab. 3)). 

In the external evaluation via EvaSys^®^ the workshop series was evaluated with the overall mark “good” (N=62; RR=19.3%). Through the free text analysis after the series of lectures and practical training on the topic of “hygiene” it became apparent that for a large number of students the importance of hygiene changed positively and the perspective of patient safety through hygienic prevention measures has intensified. Through the exercises for working in a manner which prevents infection the connection between hygiene and clinical work was made clear to the students. 

The purpose of workshop evaluation is often unclear to students [17]. The awareness of didactic or contentual changes is difficult for the students to comprehend as the improvements in teaching are usually first implemented in the following semesters. This also explains the response rates of external evaluation from 2015 in the Introduction to Clinical Medicine (ICM) (cf. table 2 (Tab. 2); RR=23%) and the series of lectures. The growing response rate in the Introduction to Clinical Medicine (ICM) in 2016 can be explained by the efforts of the teaching team. The participants in the workshop were informed that it involves a teaching project which has to be extensively evaluated for the permanent integration in the medical study curriculum. 

## Discussion

Hygiene never takes place in isolation in everyday clinical practice and contributes to the implementation of patient safety in accordance with guidelines. In particular it concerns a core competence, which must be routinely performed during medical activities. In order to achieve this it is essential that the students reflect on the connection between hygiene and other specialist fields at an early stage and it is practised independently. Through the implementation of the Introduction to Clinical Medicine (ICM) course “OT training – avoiding errors in the OT” (2015) and the restructuring of the series of lectures and practical training on the topic of “hygiene” (2017) the teaching team of the Institute of Hygiene, Hospital Epidemiology and Environmental Health succeeded in achieving this type of connection and a change of perspective. Through independently working on the various hygiene-relevant issues and through practice, it enables their own actions and behaviour in patient care to be rethought and questioned. As a result the students are made aware of working in a manner which prevents infections and are also able to recognise risks to patient safety and rectify these risks as quickly as possible in their subsequent working life. Even though the students have learned for themselves to implement examination-relevant learning objectives as a matter of priority, as part of these hygiene workshops in the pre-clinical and in the clinical section overriding objectives are pursued which the students take into account in terms of patient safety: falling infection rates, the avoidance of complications through postoperative wound infections, reduction of the amount of time spent in hospital by the patients and optimisations with regard to health economics. Through the curricular changes in medical study the students are taught practical skills which are important for working in a manner which prevents infections in subsequent everyday clinical practice [18] and for which they develop a personal sense of responsibility with regard to hygiene, which is desirable with regard to the current data situation. In a survey of the National Reference Center (NRZ) amongst qualified hygiene personnel the responsibility for the prevention of NI is predominantly viewed as being that of hygiene personnel themselves (94%) [19]. However in the view of the authors infection prevention should be an action for which you are individually responsible and a daily task of all employees in patient care. 

Personal experiences as well as the reports of students and colleagues who perform clinical work have shown that for the training of students as part of practical training/clinical clerkships in everyday clinical practice there is often insufficient time available due to the heavy workload and therefore some aspects in terms of patient safety often fall by the wayside. With the knowledge of this problem it appears necessary to integrate the content on infection prevention specifically in a practical context in workshops and take into account a consequential approach [18]. Therefore a hygiene refresher just before the beginning of the 10^th^ semester can be useful in the long-term. Although the hitherto envisaged curriculum of medical study in Leipzig does not provide for any further connection or repetition from the point of view of infection prevention and other specialist fields (see figure 1 (Fig. 1)), a continuous focus on hygiene in student education is nevertheless recommended in order to make a long-term contribution to increasing patient safety in the healthcare sector. 

In addition the restructuring of the workshops is also communicated internally at the University Hospital Leipzig as part of a feedback discussion with the operation management, in order to also receive feedback about the extent to which students who are “hygienically fitter” behave in a safer and hygienically correct manner in the operation theatre. With regard to the observed effects it is not currently possible to make any valid assertions. In order to strengthen the validity and transferability of the acquired insights the self-developed instruments (questionnaire and personal evaluation) should be adapted in future with a critical appraisal of methods.

## Conclusions

Investment in teaching is sensible for two reasons: students who are better hygienically-trained work better in subsequent practice with regard to the prevention of infection and afterwards receive in everyday clinical working life through further training in hygiene a “fine-tuning” with regard to infection prevention and consequently in terms of patient safety.

## Competing interests

The authors declare that they have no competing interests. 

## Supplementary Material

Standardised short questionnaire for the Introduction to Clinical Medicine (ICM) workshop “OT Training – avoiding errors in the OT” (since 2015)

Internal evaluation for the practical stations of the practical training series “hygiene” (since 2017)

## Figures and Tables

**Table 1:**
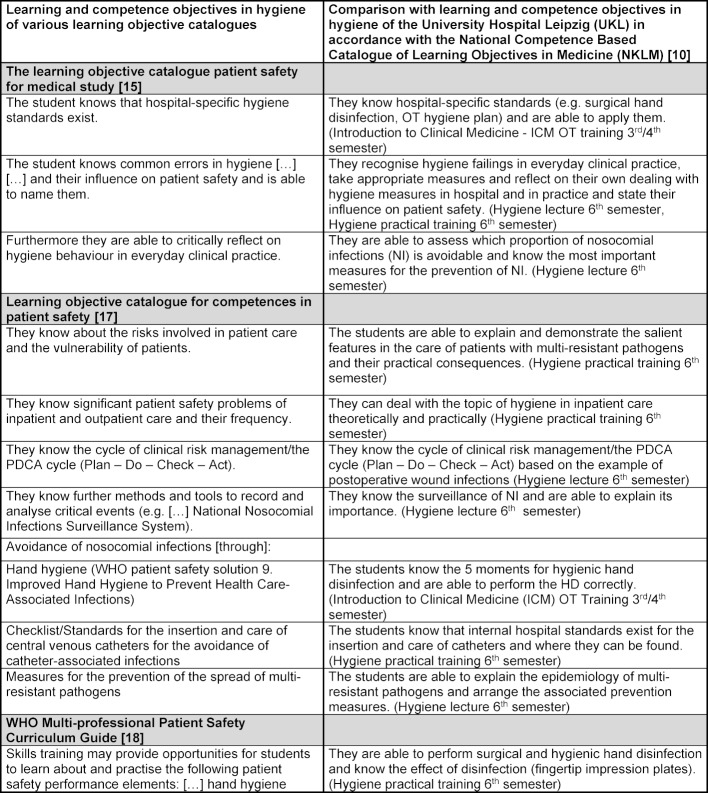
Comparison of learning objectives of various learning objective catalogues (with regard to patient safety) and learning and competence objectives of the Institute of Hygiene, Hospital Epidemiology and Environmental Health, University Hospital Leipzig (UKL)

**Table 2:**
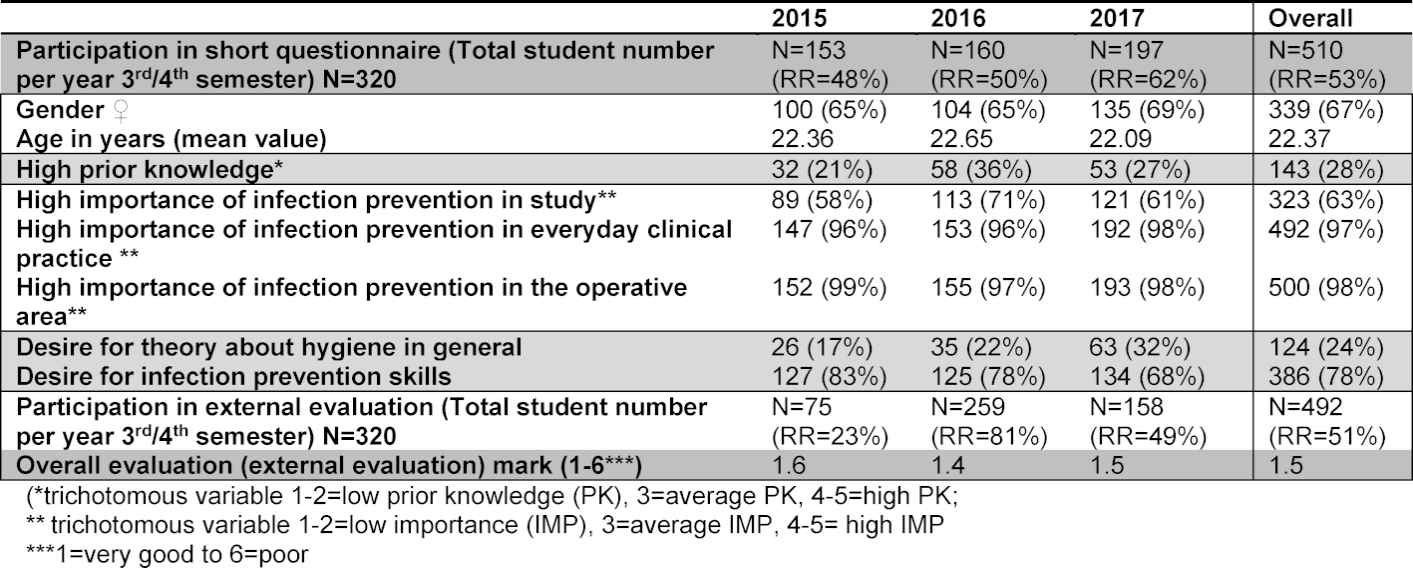
Evaluation of short questionnaire for the Introduction to Clinical Medicine (ICM) course “OT training – avoiding errors in the OT”

**Table 3:**
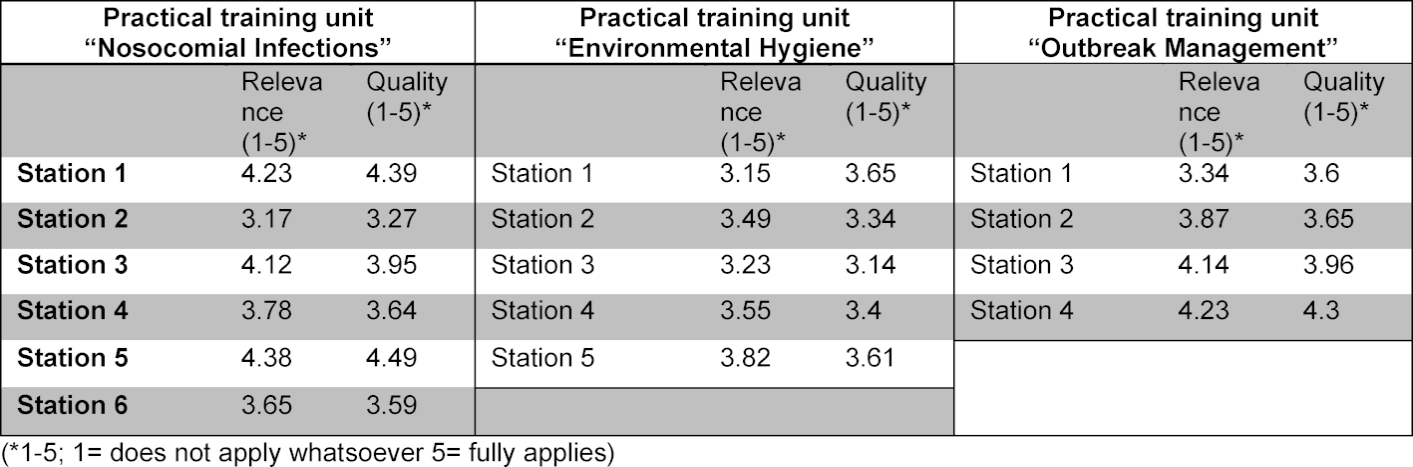
Internal evaluation results of the hygiene practical training 2017 with regard to relevance and quality; representation of the mean values
