# Supplementary material for: Hygiene in medical education – Increasing patient safety through the implementation of practical training in infection prevention
Source: GMS J Med Educ. 2019 Mar 15;36(2):Doc15. doi: 10.3205/zma001223 (PMC6446469; doi:10.3205/zma001223)
Supplement: Standardised short questionnaire for the Introduction to Clinical Medicine (ICM) workshop “OT Training – avoiding errors in the OT” (since 2015) [file JME-36-2-15-s-001.pdf]

Institute of Hygiene, Hospital Epidemiology and Environmental Health
Prof. Dr. med. Iris F. Chaberny

Introduction to Clinical Medicine (ICM) "OT Training"

Gender: ☐♀ ☐♂ **Age:** __________ **years** **Semester:** __________

What importance does the specialist area of infection prevention have for you in your study?

No importance whatsoever		Medium importance		Extremely high importance
☐1	☐2	☐3	☐4	☐5

What importance should infection prevention have fundamentally in everyday clinical practice?

No importance whatsoever		Medium importance		Extremely high importance
☐1	☐2	☐3	☐4	☐5

What importance should infection prevention have fundamentally in the operative area?

No importance whatsoever		Medium importance		Extremely high importance
☐1	☐2	☐3	☐4	☐5

Your prior knowledge on the topic of *hospital hygiene and infection prevention* is:

Very low	Low	Average	High	Very high
☐1	☐2	☐3	☐4	☐5

Which objectives do you associate with participation in the Introduction to Clinical Medicine (ICM) – OT Training:

-
-
-
-
-

Many thanks for your participation
