# Supplementary material for: Hygiene in medical education – Increasing patient safety through the implementation of practical training in infection prevention
Source: GMS J Med Educ. 2019 Mar 15;36(2):Doc15. doi: 10.3205/zma001223 (PMC6446469; doi:10.3205/zma001223)
Supplement: Internal evaluation for the practical stations of the practical training series “hygiene” (since 2017) [file JME-36-2-15-s-002.pdf]

Appendix 2: Internal evaluation for the practical stations of the practical training series “hygiene” (since 2017)

Institute of Hygiene, Hospital Epidemiology and Environmental Health
Prof. Dr. med. Iris F. Chaberny

Evaluation Practical Training Hygiene 2017

Dear Students,

Over the past year your fellow students have expressed the desire for more practical relevance with regard to hygiene in this series of workshops, therefore the concept of the practical training in hygiene has been completely revised. So that we are able to further optimise the practical training in hygiene, we ask you for additional feedback about the workshop. The answers to the questions are anonymised and cannot be attributed to you. Many thanks for your participation!

Gender: □♀ □♂ Age: __________ years Semester: __________

	Very low		Average		Very high
My prior knowledge on the topic “hygiene” **before** the workshop was	○	○	○	○	○
What importance did the topic “hygiene” have for you **before** the workshop	○	○	○	○	○

HYGIENE 1

	Does not apply whatsoever		Applies in part		Fully applies
I found the content of the “Surgical hand disinfection” station interesting.	○	○	○	○	○
I found the “Surgical hand disinfection” station well-implemented with regard to practical relevance.	○	○	○	○	○
I found the content of the “Campaign for infection prevention” station interesting.	○	○	○	○	○
I found the “Campaign for infection prevention” station well-implemented with regard to practical relevance.	○	○	○	○	○
I found the content of the “Clinical case” station interesting.	○	○	○	○	○
I found the “Clinical case” station well-implemented with regard to practical relevance.	○	○	○	○	○
I found the content of the “Dealing with medication” station interesting.	○	○	○	○	○
I found the “Dealing with medication” station well-implemented with regard to practical relevance.	○	○	○	○	○
I found the content of the “Sterile gloves” station interesting.	○	○	○	○	○
I found the “Sterile gloves” station well-implemented with regard to practical relevance.	○	○	○	○	○
I found the content of the “Dealing with PPE” station interesting.	○	○	○	○	○
I found the “Dealing with PPE” station well-implemented with regard to practical relevance.	○	○	○	○	○
**What is your final evaluation of Hygiene 1?**	○ 1	○ 2	○ 3	○ 4	○ 5

HYGIENE 2

	Does not apply whatsoever		Applies in part		Fully applies
I found the content of the “Taking a drinking water sample” station interesting.	○	○	○	○	○
I found the “Taking a drinking water sample" station well-implemented with regard to practical relevance.	○	○	○	○	○
I found the content of the “Interpretation of findings of an airborne germ sample” station interesting.	○	○	○	○	○
I found the “Interpretation of findings of an airborne germ sample” station well-implemented with regard to practical relevance.	○	○	○	○	○
I found the “Interpretation of findings of a drinking water sample” station interesting with regard to content.	○	○	○	○	○
I found the “Interpretation of findings of a drinking water sample” station well-implemented with regard to practical relevance.	○	○	○	○	○
I found the “Airborne germ sample using the example of mould” station interesting with regard to content.	○	○	○	○	○
I found the “Airborne germ sample using the example of mould” station well-implemented with regard to practical relevance.	○	○	○	○	○
I found the “Notifiable diseases” station interesting with regard to content.	○	○	○	○	○
I found the “Notifiable diseases” station well-implemented with regard to practical relevance.	○	○	○	○	○
**What is your final evaluation of Hygiene 2?**	○ 1	○ 2	○ 3	○ 4	○ 5

HYGIENE 3

	Does not apply whatsoever		Applies in part		Fully applies
I found the content of the “Hygiene training” station interesting.	○	○	○	○	○
I found the “Hygiene training” station well-implemented with regard to practical relevance.	○	○	○	○	○
I found the “Initiative vaccination” station interesting with regard to content.	○	○	○	○	○
I found the “Initiative vaccination” station well-implemented with regard to practical relevance.	○	○	○	○	○
I found the “Isolation measures and MRP” station interesting with regard to content.	○	○	○	○	○
I found the “Isolation measures and MRP” station well-implemented with regard to practical	○	○	○	○	○

Appendix 2: Internal evaluation for the practical stations of the practical training series "hygiene" (since 2017)

relevance.					
I found the "Insertion of peripheral venous catheter" station interesting with regard to content.	❍	❍	❍	❍	❍
I found the "Insertion of peripheral venous catheter" station well-implemented with regard to practical relevance.	❍	❍	❍	❍	❍
I found the "Virtual station plan" station interesting with regard to content.	❍	❍	❍	❍	❍
I found the "Virtual station plan" station well-implemented with regard to practical relevance.	❍	❍	❍	❍	❍
**What is your final evaluation of Hygiene 3?**	❍ 1	❍ 2	❍ 3	❍ 4	❍ 5
	Very low		Average		Very high
My knowledge **after** the workshop on the topic of "hygiene" was	❍	❍	❍	❍	❍
What importance did the topic "hygiene" have for you **after** the workshop?	❍	❍	❍	❍	❍
Comments, praise and criticism:					
